# A Method for Improving the Pose Accuracy of a Robot Manipulator Based on Multi-Sensor Combined Measurement and Data Fusion

**DOI:** 10.3390/s150407933

**Published:** 2015-04-02

**Authors:** Bailing Liu, Fumin Zhang, Xinghua Qu

**Affiliations:** State Key Laboratory of Precision Measuring Technology and Instruments, Tianjin University, Tianjin 300072, China; E-Mails: liubailing@tju.edu.cn (B.L.); quxinghua@tju.edu.cn (X.Q.)

**Keywords:** pose accuracy, multi-sensor data fusion, visual sensor, series robot

## Abstract

An improvement method for the pose accuracy of a robot manipulator by using a multiple-sensor combination measuring system (MCMS) is presented. It is composed of a visual sensor, an angle sensor and a series robot. The visual sensor is utilized to measure the position of the manipulator in real time, and the angle sensor is rigidly attached to the manipulator to obtain its orientation. Due to the higher accuracy of the multi-sensor, two efficient data fusion approaches, the Kalman filter (KF) and multi-sensor optimal information fusion algorithm (MOIFA), are used to fuse the position and orientation of the manipulator. The simulation and experimental results show that the pose accuracy of the robot manipulator is improved dramatically by 38%∼78% with the multi-sensor data fusion. Comparing with reported pose accuracy improvement methods, the primary advantage of this method is that it does not require the complex solution of the kinematics parameter equations, increase of the motion constraints and the complicated procedures of the traditional vision-based methods. It makes the robot processing more autonomous and accurate. To improve the reliability and accuracy of the pose measurements of MCMS, the visual sensor repeatability is experimentally studied. An optimal range of 1 × 0.8 × 1 ∼ 2 × 0.8 × 1 m in the field of view (FOV) is indicated by the experimental results.

## 1. Introduction

Since the first demonstration by Devol *et al.* in 1956, robots have been widely exploited in many fields, such as spraying, painting, spot welding, sealing, parts picking and other operations [1]. A fine accuracy in terms of the robot manipulator pose is mostly required in recent applications of industrial robots. The pose accuracy is defined precisely by ISO 9283 (1998) [2] as the deviation that occurs between the required and attained poses and the variance of the attained poses in a number of repetitions [1].

Considerable research for many years has been done about the improvement of the pose accuracy of robots. The kinematics errors refer to the differences between the kinematics parameters of a robot and its nominal values because of the manufacturing and assembly tolerance. Cost restriction aside, the kinematics calibration is an effective method to improve the absolute accuracy of robots [3]. A number of research of the kinematics calibration has been presented, e.g., Denavit and Hartenberg (1957) proposed a D-Hmodel that provided the basis for the kinematics calibration. Further, Hayati (1983) presented a revised D-H model for proposing a linear model, which related the parameter errors to the end-effector positioning error of the serial robot directly. However, these methods have limitations. For example, due to both the geometric errors and non-geometric errors, the kinematics model used in the robot controller cannot accurately describe the kinematics transformation of the actual robot. This will result in a large positioning inaccuracy [2]. Moreover, the calibration is usually performed off line, but the kinematics parameter errors often change with the load or environment variation. Therefore, the online and independent measurement is indispensable to improve the pose accuracy.

To avoid these disadvantages, some researchers begun trying to improve the pose accuracy from the perspective of the robot external measurement. They obtain the robot pose through external sensors to directly monitor the tool, workpiece and manipulator instead of considering the kinematics parameters and the influence caused by the environment. Frank S. Cheng (2007) proposed a method of robot cell calibrations to recover the accuracy of the originally defined robot tool-center-point (TCP) positions by employing a precise external sensor measuring system [4]. Hans de Ruiter (2008) presented a 3D-model-based computer-vision method for tracking the full six DOF pose of a rigid body in real time via a combination of the textured model projection and the optical flow [5]. Kaijen Hsiao (2011) applied a robot hand with tactile sensors, to localize the object on a table and ultimately achieved a target placement [6]. Qu Weiwei (2011) presented a closed-loop tracking system based on a laser sensor to reduce the relative pose error of the robot to less than 0.2 mm and ±1″ in the robot-aided aircraft assembly drilling process [7]. Guanglong Du (2014) presented an online robot self-calibration method that utilized a position sensor to obtain the position of the manipulator and an inertial measurement unit (IMU) to obtain the orientation of the manipulator in real time [3]. However, these methods also have limitations. The traditional vision-based methods utilized to calibrate a robot require the special complex steps, such as the camera calibration, corner detection and laser alignment. The laser-based methods require a large and open-sided space, and the laser beam is easily sheltered during the motion of the robot manipulator. These procedures are inconvenient, time consuming and infeasible for some applications. Therefore, in this paper, we develop a novel and flexible pose measurement system of a robot based on a visual sensor and an angle sensor. This is a quick and efficient method to improve the pose accuracy through fusing the redundant data of the multi-sensor. Our method does not require the complex solution of the kinematics parameter equations and the complicated procedures of the traditional vision-based methods, such as the camera calibration, corner detection and laser alignment. Moreover, this method is not influenced by the load and environmental variations with the online measurement. Those characteristics make the robot processing more autonomous, efficient and accurate.

In this work, we construct the flexible pose measurement system by a dynamic three-dimensional photogrammetry system, a high precision digital inclinometer and a six DOF series robot, which is rarely seen in similar research, so far. It is generally known that the absolute pose accuracy of the robot is worse than its repeatability accuracy. Fortunately, the combination of the high-precision sensors can improve the absolute pose accuracy. In this paper, as a non-contact sensor, the photogrammetry is an accurate technique that is widely used in industrial settings, yielding a measurement precision up to 1:200,000 [8]. The angle sensor, the inclinometer, has a high sensitivity to minor variations of the angle as small as 0.01°. Together with the high-precision encoding of the robot itself, the combination of all three sensors could compensate for each other to achieve a higher pose accuracy ultimately. The multi-sensors will generate a mass of data. For fusing the redundant data, this paper presents two kinds of data fusion methods, the Kalman filter fusion method (KF) and multi-sensor optimal information fusion algorithm (MOIFA), which are modeled and applied in this research.

Besides the factor of the robot itself, the measuring error of the visual sensor also can cause the inaccuracy of results. Therefore, we take the repeatability error of the visual sensor as a research object, and we find an optimal field of view (FOV) of the photogrammetry system in which its repeatability accuracy is the best.

## 2. Method for the Improvement of the Pose Accuracy of the Robot

### 2.1. System Constitution

**Figure 1:**
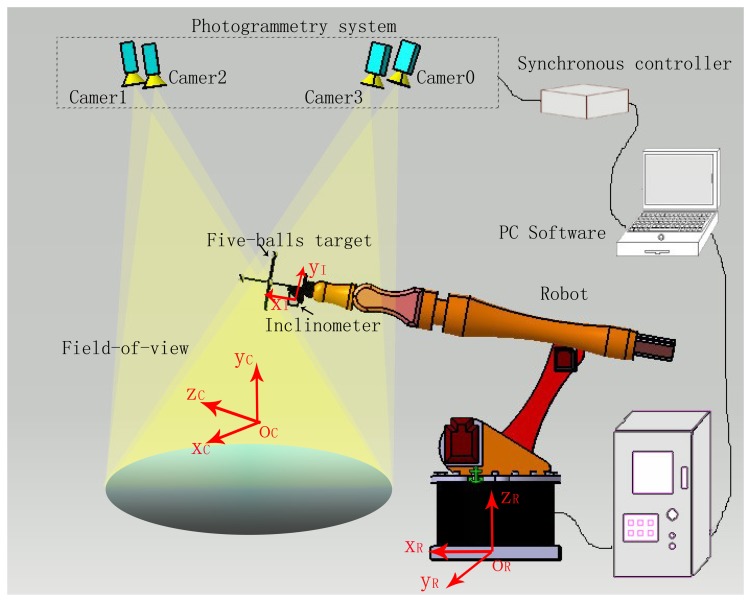
The schematic diagram of the multiple-sensor combination measuring system (MCMS).

The conventional robot system cannot decrease the pose error at the control level due to the open-loop control architecture and simplified control laws. Instead of open-loop robot systems, the multiple-sensor combination measuring system (MCMS) presented in this paper sets up a closed-loop measurement system to improve the pose accuracy of the robot. As shown in Figure 1, the described system is made up of a series robot, an industrial photogrammetry system, a digital inclinometer and the PC software. As we know, the excellent measurement performance is achieved through the use of highly sophisticated components. In this paper we adopt a 6 DOF robot, whose repeatability accuracy is 10^−3^ mm. This robot is offered by KUKA Co., Ltd. (Shanghai, China) and its model is KP 5 arc. We also choose a high precision industrial three-dimensional photogrammetry system to dynamically track and measure the robot pose in real time. The photogrammetry system is composed of 4 motion-sensitive CCD cameras set on top of the robot. We also adopt a high accuracy digital inclinometer LE-30, whose accuracy is 0.01°. The inclinometer can rapidly measure both the angle of the pitch and yaw at a 20-ms frequency, and it can be set up on the robot manipulator to measure the attitude angle in real time. However, only possessing highly sophisticated instruments is not enough; the pose accuracy will be improved through fusing the redundant data of the sensors. These data should be converted by the coordinate transformation matrix previously.

### 2.2. Method of Data Fusion

As is well known, the primary aim of the sensor fusion is to improve the accuracy by using the redundant information gathered from multiple sources [9]. Since the construction of the MCMS, the sensor fusion is suitable to apply in this system. Currently, a number of different types of data fusion methods are being used in industry, such as the Wiener filter, constrained least squares filter, *α-β* filter, *α*-*β*-*γ* filter, Kalman filter [10], linear minimum variance fusion algorithm, *etc*. The Kalman filter (KF), first proposed in 1960 by Kalman [11], has been successfully used in the Apollo moon flight and C-5A aircraft navigation systems. Walker and Harries [12] improved the system robustness and adaptability in the mobile robot area through KF and multi-sensor fusion. The multi-sensor optimal information fusion algorithm in the linear minimum variance sense (MOIFA) is a geometric fusion method that was developed by Nakamura [13] and has been enhanced by Elliot *et al.* [14]. A demonstration of its use can be seen in [15], where the method was applied to an optical encoder and a camera sensor [16]. The two methods allow improving the fusion accuracy significantly, so we choose both of them as the methods of data fusion in this paper. We will summarize them respectively in Sections 2.2.1 and 2.2.2.

#### 2.2.1. Kalman Filter

The Kalman filter solves the optimal linear filtering problems on the basis of a minimum mean square error method. The present value of the signal can be calculated according to the prior predicted value and the latest observation data. The Kalman filter predicts the value through a group of state equations and recursive methods. This recursive solution usually is expressed in the form of the predicted value. The following Equations (1)–(5) are the recursion formulas of the Kalman filter:

(1) X(k)=X(k/k−1)+H(k)[Y(k)−C(k)X(k/k−1)]

(2) X(k/k−1)=A(k)X(k−1)

(3) $$H\left(k\right)=p\left(k/k-1\right)C{\left(k\right)}^{T}{\left[C\left(k\right)p\left(k/k-1\right)C{\left(k\right)}^{T}+R\left(k\right)\right]}^{-1}$$

(4) $$p\left(k/k-1\right)=A\left(k\right)p\left(k-1\right)A{\left(k\right)}^{T}+B\left(k\right)Q\left(k\right)B{\left(k\right)}^{T}$$

(5) p(k)=[I−H(k)C(k)]p(k/k−1)

where *X* (*k*) is a multi-dimensional state vector *i.e.*, the predicted value at time *k* of a single sensor. *C* (*k*) is an observation vector. *A* (*k*) and *B* (*k*) are the transfer matrices determined by the system. *Y* (*k*) is the observation value of a single sensor. *Q* (*k*) is the system noise matrix, and *R* (*k*) is the measurement noise matrix. The statistics features *E* [*X*(0)] and *var* [*X*(0)] of the initial state *X*(0) are known as *X*(0) = *E*[*X*(0)] = *μ*_0_ and *p*(0) = *E*[*(X*(0) − *E*(*X*(0)))^2^] = var[*X*(0)].

Substituting *p*(0) into Equation (4), we obtain *p*(1/0). Substituting *p*(1/0) into Equation (3), we obtain *H* (1). Substituting *H* (1) into Equation (1), we obtain *X* (1) in the condition of the minimum mean square error. At the same time, substituting *p* (1/0) into Equation (5), we obtain *p* (1). Then, we obtain *p* (2/1) by *p* (1), *H* (2) by *p* (2/1) and *X* (2) by *H* (2), the as same as above, and so on. Therefore, the predicted value at time *k* can be calculated.

In this paper, we only take into account the position accuracy of the robot. Two sensors, the photogrammetry system and the series robot, are utilized. *Y*^1^(*k*) is the observation value at time k of the photogrammetry system. *Y*^2^(*k*) is the observation value at time k of the robot, and *X*^1^(*k*) is the predicted value at time *k* of the photogrammetry system. *X*^2^(*k*) is the predicted value at time *k* of the robot. *X^f^*(*k*) is fused by *X*^1^(*k*) and *X*^2^(*k*) with the weighting matrix W, which is determined by the typical accuracy values of the measuring instruments. Following is the principle of data fusion:

*X^i^* is the three-dimensional coordinate of the predicted value with the *i*-th sensor. It usually can be expressed by means of a function of *θ* as *X^i^* = *f*(*θ^i^*), and *θ* is a multi-dimensional vector. A predicted value with additive noise can be represented as *X^i^* = *f*(*θ^i^*+*δθ^i^*), where *δθ^i^* represents the additive noise. Equation (6) is deduced by Taylor expanding *X^i^* and neglecting the quadratic term.

(6) $$X^{i}=f\left(\theta^{i}\right)+J\left(\theta^{i}\right)\delta \theta^{i}$$

where *J*(*θ^i^*) is the Jacobian matrix of the *i*-th sensor, $J\left(\theta^{i}\right)=\frac{\partial X^{i}}{\partial \theta^{i}}.$

Assuming a Gaussian distribution for the noise gives $E\left[\delta \theta^{i}\right]≜\bar{\delta \theta^{i}}=0$. Combining Equation (6), the mean and covariance of *X^i^* are:

(7) $$E\left[X^{i}\right]≜\bar{X^{i}}=f\left(\theta^{i}\right)$$

(8) $$V\left[X^{i}\right]≜E\left[\left(X^{i}-\bar{X^{i}}\right){\left(X^{i}-\bar{X^{i}}\right)}^{T}\right]=E\left[J^{i}\delta \theta^{i}\delta \theta^{i^{T}}J^{i^{T}}\right]=J^{i}Q^{i}J^{i^{T}}$$

where *Q^i^* is the covariance matrix of *δθ^i^*.

The weight matrix is defined as *W* = (*W*^1^*W*^2^…*W*^n^), and n is the number of the sensors. The fusion value at time *k* combines multiple measurements by the weighted average:

(9) $$X\left(k\right)=\sum_{i=1}^{n}W^{i}X^{i}\left(k\right)$$

where *W* ∈ 3 × 3*n*. *W^i^* is the weighting matrix of the *i*-th sensor. It is assumed that $\bar{X^{i}}=\bar{X}$, *i.e.*, all measurement instruments are properly calibrated. Using Equations (9) and (7), the fused mean value becomes:

(10) $$E\left[X\left(k\right)\right]=\sum_{i=1}^{n}W^{i}E\left[X^{i}\left(k\right)\right]=\sum_{i=1}^{n}W^{i}\bar{X^{i}}\left(k\right)=\left(\sum_{i=1}^{n}W^{i}\right)\bar{X}\left(k\right)$$

since,

(11) $$E\left[X\left(k\right)\right]=\bar{X}\left(k\right)$$

then, $\overset{n}{\underset{i=1}{\text{∑}}}W^{i}=I$, where **I** is the identity matrix.

Using Equations (6)–(11), the covariance matrix becomes:

(12) $$\begin{array}{l} V\left[X\left(k\right)\right]=E\left[\left(X\left(k\right)-\bar{X}\left(k\right)\right){\left(X\left(k\right)-\bar{X}\left(k\right)\right)}^{T}\right]\\ =E\left[\left(\overset{n}{\underset{i=1}{\text{∑}}}W^{i}X^{i}\left(k\right)-\overset{n}{\underset{i=1}{\text{∑}}}W^{i}\bar{X^{i}}\left(k\right)\right){\left(\overset{n}{\underset{i=1}{\text{∑}}}W^{i}X^{i}\left(k\right)-\overset{n}{\underset{i=1}{\text{∑}}}W^{i}\bar{X^{i}}\left(k\right)\right)}^{T}\right]\\ =\overset{n}{\underset{i=1}{\text{∑}}}W^{i}J^{i}Q^{i}J^{iT}W^{iT} \end{array}$$

*W^i^* can be solved by means of Lagrange's method. The solving process is detailed in [13]. The weighting matrix is given to be:

(13) $$W^{i}={\left\{\sum_{i=1}^{n}\left(J^{i}Q^{i}J^{iT}\right)\right\}}^{-1}{\left(J^{i}Q^{i}J^{iT}\right)}^{-1}$$

In this paper, *W*^1^ and *W*^2^ represent the weighting matrices of the photogrammetry system and the series robot separately. According to Equation (9), the fused result at time *k* is:

(14) $$X^{f}\left(k\right)=W^{1}X^{1}\left(k\right)+W^{2}X^{2}\left(k\right)$$

#### 2.2.2. Multi-Sensor Optimal Information Fusion Algorithm

The optimum fused value of the spatial coordinates for the robot position can be calculated as shown in Equation (15). “The optimum value” means the minimum variance unbiased estimate of the fused result *X̂*.

(15) $$\hat{X}=\sum_{i=1}^{n}W^{i}X^{i}$$

where *i* is the number of measuring instruments. The weighting matrix is shown to be:

(16) $$W^{i}={\left\{\sum_{i=1}^{n}\left(J^{i}Q^{i}J^{iT}\right)\right\}}^{-1}{\left(J^{i}Q^{i}J^{iT}\right)}^{-1}$$

where *Q^i^, J^i^* are the covariance matrix and the Jacobian matrix of the *i*-th measuring instrument. In this paper, the covariance matrices of the photogrammetry system and robot are shown below:

(17) $$Q^{c}=\left[\begin{array}{lll} {\delta_{xc}}^{2} & & \\ & {\delta_{yc}}^{2} & \\ & & {\delta_{zc}}^{2} \end{array}\right]\;Q^{r}=\left[\begin{array}{lll} {\delta_{xr}}^{2} & & \\ & {\delta_{yr}}^{2} & \\ & & {\delta_{zr}}^{2} \end{array}\right]$$

where (*δ_xc_*,*δ_yc_*,*δ_zc_*), (*δ_xr_*,*δ_yr_*,*δ_zr_*) are three components of the typical accuracy values the photogrammetry system and of the robot. Substituting *Q^c^* and *Q^r^* into Equations (15) and (16), we can obtain the optimum fused result.

The covariance of *X^i^* is given to be:

(18) $$V^{i}\left(X\right)=J^{i}Q^{i}J^{iT}$$

The fused covariance of *X̂* is given below:

(19) $$V\left(X\right)={\left\{\sum_{i=1}^{n}{\left(J^{i}Q^{i}J^{iT}\right)}^{-1}\right\}}^{-1}$$

It obviously can be deduced from Equation (19) that *V*(*X*)^−1^ > *V^i^*(*X*)^−1^; then *V*(*X*) < *V^i^*(*X*). This proves that the fusion accuracy is better than the local accuracy.

## 3. Experiments and Discussion

To improve the accuracy of a robot manipulator, there are two steps in this paper. Firstly, the accuracy of a robot manipulator can be improved through the calibration for the kinematics parameters of the robot by the photogrammetry system. In addition, through calibrating the kinematics parameters of the robot, we can obtain a transformation relationship between the coordinate system of the photogrammetry system and robot. This makes the base coordinate system of the robot be a local unified coordinate system. Secondly, using the pose data of the calibrated robot and the online measurement of the multi-sensor combined measurement system (MCMS), three kinds of measurement data can be obtained. They are from the calibrated robot, photogrammetry system and inclinometer. The result can be improved by fusing these redundant data through KF and MOIFA. Therefore, four experiments are designed in this paper. Firstly, as an important part in MCMS, since the photogrammetry system directly monitors the robot pose, the accuracy of the photogrammetry system is crucial to the whole measurement system. The measurement errors of the photogrammetry system often appear to be due to the distortion on the edge of the FOV and the mistaken identity to the target. Therefore, it is necessary to research the repeatability accuracy of the photogrammetry system in its FOV, which is detailed in Section 3.1. Secondly, to improve the pose accuracy of the robot, the primary work is the calibration of the robot. Therefore, the experiment using the photogrammetry system to calibrate the robot is designed in Section 3.2. Thirdly, to observe the effect of the two data fusion methods, we design a simulation in Section 3.3. At last, in Section 3.4, a lab experiment is designed for verifying the result of the simulation. In this paper, all accuracy values (simulations and measurements) are given through the “three-sigma rule”, which is a method of eliminating the gross error by thrice the standard error in the theory of errors.

### 3.1. Repeatability Precision of Photogrammetry System

**Figure 2:**
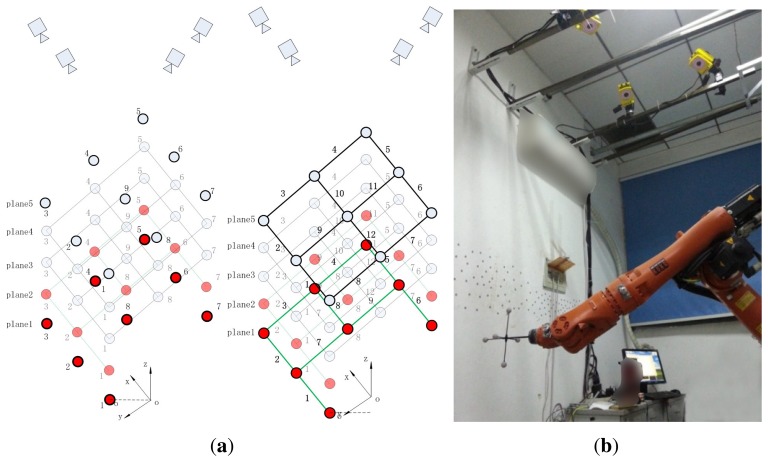
(**a**) Experimental principle of repeatability precision of the photogrammetry system; (**b**) the image of the experimental field.

One of the ways to improve the photogrammetry system accuracy is to search for the optimal range of the FOV. As shown in Figure 2a, in order to test the accuracy of the photogrammetry system in the FOV, an experiment is designed as follows. The space of 1 × 0.8 × 1 m is divided into five planes from bottom to top, each of which contains 8∼9 points. Then, 9∼12 lines are formed through connecting the adjacent points. We move the robot manipulator to these points sequentially, and the photogrammetry system measures the coordinates of each point five times. In this paper, the laser tracker offered by FARO Co., Ltd. measures all points three times, and the result is used as the reference value. The FARO Xi laser tracker in the lab, whose uncertainty of the absolute distance meter (ADM) is 10 μm + 1.1 μm/mL, has been verified by the National Metrology Institute of China (NIM CDjx2008-0782). The measurement of the lines is similar as the points. The image of the experimental field is shown in Figure 2b.

#### 3.1.1. Results of the Repeatability Precision of the Photogrammetry System

As shown in Table 1, the standard deviations of the position for 43 points are calculated by five groups of data, where δ_x_,δ_y_,δ_z_ are the standard deviations of three dimensions. Due to space limitations, we extract the data of Planes 1 and 5. δ_d_ is the compound standard deviation of δ_x_,δ_y_,δ_z_.

**Figure 3:**
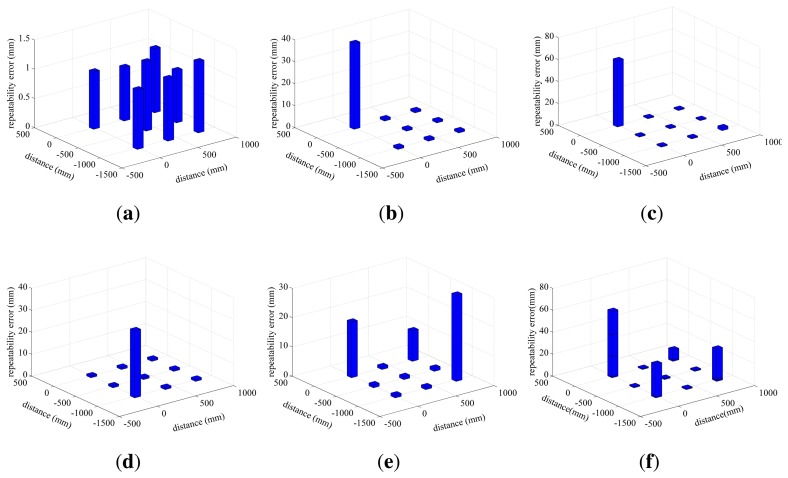
The histograms of the error of five planes and the merged error of all planes.

In order to show the distribution of the repeatability error in the FOV of the photogrammetry system, the histograms of the standard deviation are drawn in Figure 3. Figure 3a–e shows the error of Planes 1∼5, and Figure 3f shows the merged errors of all planes.

Some phenomena are observed in Figure 3. Firstly, the maximum error appears at the corner of each plane, such as Points 1, 3, 5, 7. Their merged errors are much larger than the other points, as shown in Figure 3. Except for these cornered points, the errors of the rest of the points are almost similar. Secondly, with the decreasing of the distance between the plane and the camera, the values and the number of the errors increase. Thirdly, the errors in the direction of x and z are smaller than y.

In this paper, TENYOUNfull body motion capture 3DMoCap-GC130 is used as a visual sensor. It takes more than 6 mm of prime lens as the optical lens of the camera. Its measurement range is more than 1 × 1 × 1 m. As we known, the FOV of the camera will enlarge with increasing of the photograph distance. The size of Plane 5 is 1 × 0.8 × 1 m, which can be considered to be the range close to the limitation of the measurement range. Plane 1, the length size of which is 1 × 0.8 × 2 m, is in a reasonable measurement range. Therefore, the error of Plane 5 is the largest in all planes and that of Plane 1 is the smallest. The result in Table 1 and Figure 3 shows that the repeatability error in the range of 1 × 0.8 × 1 ∼ 2 × 0.8 × 1 m has higher accuracy. The best accuracy is in the center of the range, the average values of which are δ_x_ = 0.124 mm, δ_y_ = 0.997 mm, δ_z_ = 0.272 mm, δ_d_ = 1.045 mm.

**Figure 4:**
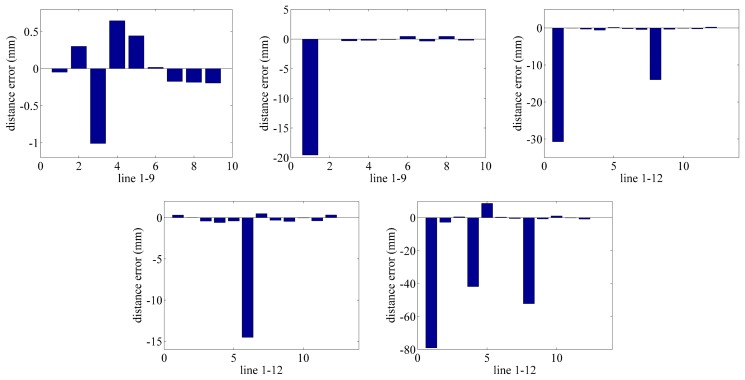
The error of the lines of five planes.

Secondly, we discuss the situation of the lines. As shown in Table 2, the standard deviations of 54 lines are calculated in comparison to the data of the laser tracker. Similarly, we select the data of Plane 1 and Plane 5. d_l_ is the length measured by the laser tracker, and d_c_ is the length measured by the photogrammetry system. Δ_d_ is the difference of the laser tracker and the photogrammetry system. The errors of the lines of each plane are drawn in Figure 4.

From Table 2 and Figure 4, we can find that the error of the lines has a similar phenomena as the points. The error of the lines increases with the decreasing of the distance between the plane and camera. The peak of the error appears in Lines 3, 4, 6, 8, which connect to the corner Points 1, 3, 5, 7. Accordingly, a conclusion can be drawn that the accuracy of the photogrammetry system is much higher in the range of 1 × 0.8 × 1 ∼ 2 × 0.8 × 1 m. The best accuracy of the lines is located in the center of the FOV, and its average value is Δd = 0.478 mm.

Therefore, the photogrammetry system can be used as the calibration instrument. The measured data also can be used as the feedback data to compensate for the errors of the robot in its effective measurement range.

### 3.2. Calibrating Method for the Robot

One of the ways to improve the pose accuracy for the robot manipulator is to calibrate the kinematics parameters of the robot. The photogrammetry system is used to monitor the pose of robot, so that it is reasonable to calibrate the robot by means of it. This paper proposes a calibrating method for the position error of the robot that is based on the D-H model. The first step of this method is to build a model of the coordinate transformation between the coordinate system of the photogrammetry system and the robot. Then, a kinematics parameter model of the robot manipulator is established according to the differential equation of the kinematics parameters error for the robot axes. The measured data of the photogrammetry system are converted into the coordinate system of the robot. Through comparing with the converted measured data, the parameters of the robot, such as the robot kinematics parameter, target installation error and transferred error of coordinate system, can be calibrated. A simple description of the main principle is shown below. The details of the calibrated method are shown in [17,18].

**Figure 5:**
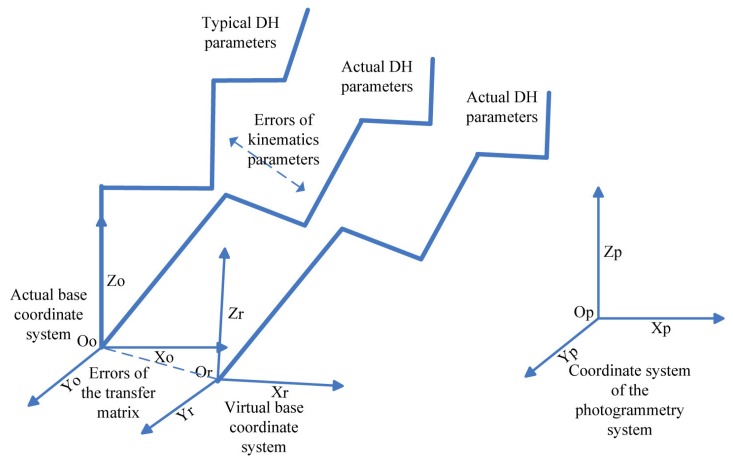
Simplified model of the robot calibration.

Figure 5 shows a simple model of the robot calibration. *O_p_X_p_Y_p_Z_p_* is the coordinate system of the photogrammetry system. *O_o_X_o_Y_o_Z_o_* is the actual base coordinate system of the robot. *O_r_X_r_Y_r_Z_r_* is the virtual base coordinate system of the robot measured by the photogrammetry system. The difference between the *O_o_X_o_Y_o_Z_o_* and *O_r_X_r_Y_r_Z_r_* is caused by the errors of the transfer matrix.

In order to obtain the position error of the robot, we must unify the coordinate systems of the photogrammetry system to the robot firstly. Assume ${\text{T}}_{p}^{r}=\left[\begin{array}{llll} r_{11} & r_{12} & r_{13} & \text{x}\\ r_{21} & r_{22} & r_{23} & \text{y}\\ r_{31} & r_{32} & r_{33} & \text{z}\\ 0 & 0 & 0 & 1 \end{array}\right]$ is the transfer matrix from the coordinate system of the photogrammetry system *O_p_X_p_Y_p_Z_p_* to the virtual base coordinate system of the robot *O_r_X_r_Y_r_Z_r_.* Rotating Axis 1 of the robot with a certain degree, the photogrammetry system can obtain a group of data. Fitting the data, we obtain the vector of the direction *z*, which is the third component of ${\text{T}}_{p}^{r}$. Similarly, the vector of the direction *x*, *i.e.*, the first component of ${\text{T}}_{p}^{r}$, can be obtained by rotating Axis 2 of the robot. Then, the vector of the direction *y*, *i.e.*, the second component of ${\text{T}}_{p}^{r}$, can be calculated by the cross-product of vector *z* and *x*. The translation vector (*x, y, z*) also can be fitted by the data.

Suppose that the error model of the transfer matrix from the *O_r_X_r_Y_r_Z_r_* and *O_o_X_o_Y_o_Z_o_* is expressed as Equation (20):

(20) $${\text{T}}_{r}^{o}=\left[\begin{array}{llll} 1 & -\delta_{z} & \delta_{y} & {\text{d}}_{x}\\ \delta_{z} & 1 & -\delta_{x} & {\text{d}}_{y}\\ -\delta_{y} & \delta_{x} & 1 & {\text{d}}_{z}\\ 0 & 0 & 0 & 1 \end{array}\right]$$

where *δ_x_*, *δ_y_*, *δ*_z_ are the errors of the rotation matrix and *d_x_*, *d_y_*, *d_z_* are the errors of the translation matrix.

In addition, the cooperation target of the photogrammetry system, which is set up at the end axis of the robot, should be considered as an additional axis, Axis 7. Therefore, the transfer matrix from Axis 6 to Axis 7 is shown in Equation (21).

(21) $${\text{T}}_{6}^{7}=\left[\begin{array}{llll} 1 & 0 & 0 & {\text{t}}_{\text{x}}\\ 0 & 1 & 0 & {\text{t}}_{\text{y}}\\ 0 & 0 & 1 & {\text{t}}_{\text{z}}\\ 0 & 0 & 0 & 1 \end{array}\right]$$

where *t_x_*, *t_y_*, *t_z_* are the translation vectors, which can be measured previously.

Therefore, the transfer matrix from the coordinate system of the photogrammetry system to the coordinate system of the robot manipulator is shown in Equation (22).

(22) $$\text{T}=\left(\sum_{i=1}^{7}{\text{T}}_{\text{i}-1}^{\text{i}}\right){\text{T}}_{r}^{o}{\text{T}}_{p}^{o}$$

Assume $B_{p}=\left[\begin{array}{llll} r_{1p} & r_{2p} & r_{3p} & p_{xp}\\ r_{4p} & r_{5p} & r_{6p} & p_{yp}\\ r_{7p} & r_{8p} & r_{9p} & p_{zp}\\ 0 & 0 & 0 & 1 \end{array}\right]$ is the pose of a certain point in the coordinate system of the photogrammetry system, where *r*_1_*_p_* ∼ *r*_9_*_p_* are the attitude parameters and *p_xp_* ∼ *p_zp_* are the position parameters. The converted pose of this point from the coordinate system of the photogrammetry system to the coordinate system of the robot manipulator $B_{r}=\left[\begin{array}{llll} r_{1r} & r_{2r} & r_{3r} & p_{xr}\\ r_{4r} & r_{5r} & r_{6r} & p_{yr}\\ r_{7r} & r_{8r} & r_{9r} & p_{zr}\\ 0 & 0 & 0 & 1 \end{array}\right]$ can be obtained by calculation of *B_r_* = *T* · *B_p_* using Equation (22). Then, the Z-Y-Z Euler angles (*ϕ*, *θ*, *ψ*), which express the attitude angles of the robot manipulator, can be obtained as shown in Equation (23).

(23) $$\{\begin{array}{l} \phi =\arctan\frac{r_{6r}}{r_{3r}}\\ \theta =\arctan\frac{\sqrt{{r_{7r}}^{2}+{r_{8r}}^{2}}}{r_{9r}}\\ \psi =\arctan\frac{r_{8r}}{-r_{1r}} \end{array}$$

The link parameters of the robot are the most significant impact factors on the position error of the robot. In the D-H model, they are the length of the link *a*, the displacement of the link *d*, the angle of rotation *α* and the link angle of each axis *θ*. In this paper, we adopt a series robot of six axes, so we can totally obtain 24 kinematics parameters Δ*a*_1∼4_, Δ*d*_1∼4_, Δ*α*_1∼4_, Δ*θ*_1∼4_. In addition, the transfer matrix from the coordinate system of the robot tool-center-point (TCP) to the end axis of the robot has nine rotation and translation error variables, as Equation (20) shows. Therefore, there are 33 parameters of the robot that need to be calibrated.

According to the distance error model of a series robot [17], the relationship of the distance error and position error is shown as Equation (24).

(24) $$\Delta l\left(i,i+1\right)=\left[\frac{x_{R}\left(i+1\right)-x_{R}\left(i\right)}{l_{R}\left(i,i+1\right)},\frac{y_{R}\left(i+1\right)-y_{R}\left(i\right)}{l_{R}\left(i,i+1\right)},\frac{z_{R}\left(i+1\right)-z_{R}\left(i\right)}{l_{R}\left(i,i+1\right)}\right]\cdot \left(d\vec{p_{i+1}}-\vec{p_{i}}\right)$$

where *i* is the number of the point on the command trajectory. *l_R_*(*i*,*i* + 1) is the distance between the point *i* and *i* + 1 on the command trajectory. (*x_R_*, *y_R_*, *z_R_*) is the position coordinate components of a certain point in the robot coordinate system *O_o_X_o_Y_o_Z_o_*. Δ*l* is the distance error, *i.e.*, the difference value between the theoretical position and practical position. In this paper, the theoretical value is obtained by the robot encoder, and the practical value is obtained by the photogrammetry system. *dp* is the vector for the position error of the robot.

Because of the impact of four link parameters of the robot, it will cause the position error for the adjacent axes of the robot ${\text{dT}}_{\text{i}-1}^{\text{i}}$, which can be expressed as Equation (25).

(25) $$dT_{i-1}^{i}=\frac{\partial T_{i-1}^{i}}{\partial \theta_{i}}\Delta \theta_{i}+\frac{\partial T_{i-1}^{i}}{\partial \alpha_{i}}\Delta \alpha_{i}+\frac{\partial T_{i-1}^{i}}{\partial a_{i}}\Delta a_{i}+\frac{\partial T_{i-1}^{i}}{\partial d_{i}}\Delta d_{i}$$

If each of the two adjacent axes are influenced by the link parameters, the transformation from the base coordinate system of the robot to the coordinate system of the robot manipulator can be expressed as Equation (26). In this paper, N = 6.

(26) $${\text{T}}_{0}^{\text{N}}+{\text{dT}}_{0}^{\text{N}}=\prod_{\text{i}=1}^{\text{N}}\left({\text{T}}_{\text{i}-1}^{\text{i}}+{\text{dT}}_{\text{i}-1}^{\text{i}}\right)=\prod_{\text{i}=1}^{\text{N}}\left({\text{T}}_{\text{i}-1}^{\text{i}}+{\text{T}}_{\text{i}-1}^{\text{i}}\Delta_{\text{i}}\right)$$

where Δ*_i_* = *T_θi_* · Δ*θ_i_* + *T_αi_* · Δ*α_i_* + *T_ai_* · Δ*a_i_* + *T_di_* · Δ*d_i_*. Additionally, *T_θi_*, *T_αi_*, *T_ai_*, *T_di_* can be obtained by the calculation of the robot kinematics parameters. Through expanding Equation (26) with a large number of simplifications and combinations, the position error of the robot manipulator can be obtained as given in Equation (27). More detail about the calculation procedures can be found in [17].

(27) $$\begin{array}{l} \Delta p={\left[d_{tx}d_{ty}d_{tz}\right]}^{T}\\ =\left[\begin{array}{cccccccccc} k_{1\theta }^{x} & k_{1\alpha }^{x} & k_{1a}^{x} & k_{1d}^{x} & k_{2\theta }^{x} & \cdots & k_{6\theta }^{x} & k_{tx}^{x} & k_{ty}^{x} & k_{tz}^{x}\\ k_{1\theta }^{y} & k_{1\alpha }^{y} & k_{1a}^{y} & k_{1d}^{y} & k_{2\theta }^{y} & \cdots & k_{6\theta }^{y} & k_{tx}^{y} & k_{ty}^{y} & k_{tz}^{y}\\ k_{1\theta }^{z} & k_{1\alpha }^{z} & k_{1a}^{x} & k_{1d}^{z} & k_{2\theta }^{z} & \cdots & k_{6\theta }^{z} & k_{tx}^{z} & k_{ty}^{z} & k_{tz}^{z} \end{array}\right].\\ {\left[\Delta \theta_{1}\Delta \alpha_{1}\Delta a_{1}\Delta \theta_{2}\cdots \Delta d_{6}\Delta t_{x}\Delta t_{y}\Delta t_{z}\right]}^{T}\\ =B_{i}\Delta q_{i} \end{array}$$

where Δ*p* is the position error of the robot manipulator. (*d_tx_*, *d_ty_*, *d_tz_*) are the Cartesian coordinate components of the position error. $B_{i}=\left[\begin{array}{lll} k_{1\theta }^{x} & \cdots & k_{tz}^{x}\\ \vdots & \ddots & \vdots \\ k_{1\theta }^{z} & \cdots & k_{1\theta }^{z} \end{array}\right]$ is the parameter matrix related to the typical position value of the robot manipulator. Δ*_qi_* = [Δ*θ*_1_ ⋯ Δ*t_z_*]*^T^* are the kinematics parameters of six-degree series robot and the translation error parameters from the coordinate system of the robot TCP to the end axis of the robot. In Equation (27), the left side is the position error at each point measured by the photogrammetry system. The right side is the kinematics errors that need to be corrected. These errors can be revised by the least squares method in the generalized inverse matrix sense.

**Figure 6:**
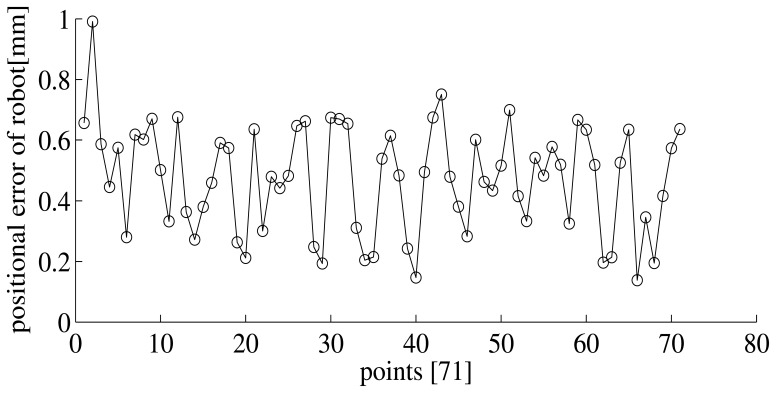
The position error of the robot after calibration.

After calibrating by the photogrammetry system, the position error of the robot can be less than 1 mm. Figure 6 shows the position error of 71 points.

### 3.3. Simulation Test of the Sensor Data Fusion Methods

In terms of the position of the robot manipulator, there are two kinds of measurement data: one is obtained from the photogrammetry system and the other one is from the encoder of the robot. We propose two sensor data fusion methods to fuse the two kinds of position data. In order to compare the two methods, a simulation test is developed in MATLAB. One-hundred random points are created in a space of 100 × 100 × 100 mm to simulate the actual positions of the robot. For the purpose of simulating the actual value, each point is mixed with an error. It follows the normal distribution, which is determined by the typical value *δ* of the measured instruments. In this paper, the typical accuracy values of the photogrammetry system are *δ_xc_* = *δ_yc_* = *δ_zc_* = 0.15 mm. The typical accuracy values of the robot are *δ_xr_* = 0.157 mm, *δ_yr_* = 0.087 mm, *δ_zr_* = 0.043 mm. The typical values are calibrated by the FARO Xi laser tracker. Then, 100 points are fused by using KF and MOIFA.

**Figure 7:**
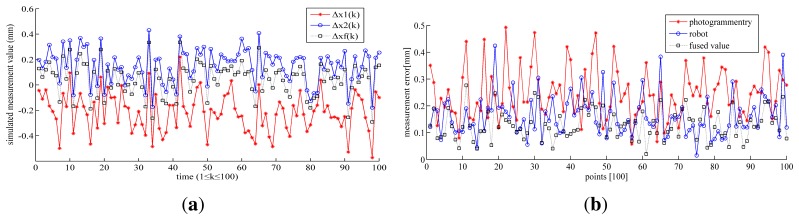
(**a**) The simulated fused error using the KF method; (**b**) The simulated fused error using the multi-sensor optimal information fusion algorithm (MOIFA).

In the first method, the simulated measured value of the photogrammetry system and robot, *Y*^1^(k) and *Y*^2^(k), are input into KF, as described in Equations (1)–(5), to obtain the estimated state variables, *X*^1^(*k* + 1) and *X*^2^(*k* + 1). Then, they are fused using the weight matrix W described in Equation (14), which is determined by the Jacobian matrices and covariance matrices of the photogrammetry system and robot. The fused error using KF is drawn in Figure 7a. In the second method, the simulated value of the photogrammetry and robot are fused as described in Equations (15)–(17). The fusion errors of MOIFA are drawn in Figure 7b.

#### 3.3.1. Results of the Simulation Test of the Data Fusion Methods

As shown in Table 3, Δ*x*_1_, Δ*x*_2_ are the estimated errors of the photogrammetry and robot after fusing by the KF method, respectively. Δ*x_f_* is the fused error. Δ*_CM_*, Δ*_RB_* are the errors of the photogrammetry and robot, respectively. Δ*_f_* is the fused error after fusing by MOIFA.

Since the photogrammetry system has a bigger typical accuracy value, the error of the photogrammetry system is bigger than that of the robot, as shown in Table 3. It is indicated in Figure 7a,b and Table 3 that either of the methods can reduce the error after the data fusion. Through calculating the data in Table 3, the error is reduced by 78.2% with KF and by 46.1% with MOIFA. As shown in Table 3, KF has smaller fused errors than MOIFA. In addition, KF can predict the state variables of next moment, which is suitable to be applied in the dynamic measurement and compensation. MOIFA can only analyze the ready-measured data. However, it will cause the hysteresis of the real-time compensation. It should be noted that the measuring range is 0∼100 mm in this experiment. KF is a linear filter, so that its fused error will be enlarged with increasing the measuring range. This will be verified in the lab experiment in Section 3.4.

### 3.4. Verified Experiment in the Lab

In order to validate the conclusion of the simulation test, a data fusion-verified experiment in lab is designed. For the purpose of measuring the pose of the robot manipulator, a five-ball target frame of the photogrammetry system and the inclinometer are set up at the end of the robot through a special fixture, as shown in Figure 1. Seventy six points are located on the surface of a 200-mm radius sphere, which is in the space of 1 × 1 × 1 m in the front of the robot. Obviously, these points must also be located in the effective measurement area of the photogrammetry system, as described in Section 3.1. For acquiring the stable data, the robot stays in each position for 7 s to offer enough measurement time for the photogrammetry system. Since the robot has been calibrated in Section 3.2, in this experiment, all data are converted into the base coordinate system of the robot. The measurement value of the FARO Xi laser tracker is used as the reference value. Then, 71 picked points are fused using KF and MOIFA.

**Figure 8:**
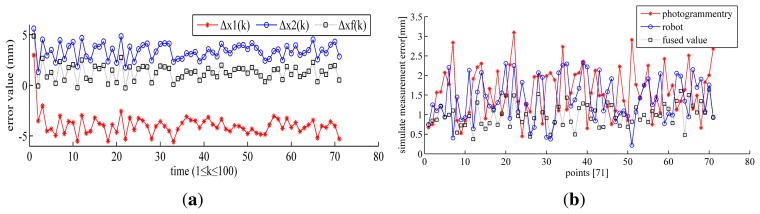
(**a**) The experimental error using KF; (**b**) the experimental error using MOIFA.

The same as the process of the simulate experiment described in Section 3.3, the fused error using KF is drawn in Figure 8a, and the fused error using MOIFA is drawn in Figure 8b.

#### 3.4.1. Result of the Verified Experiment in the Lab

The average values of the measurement error are shown in Table 4.

It is indicated in Table 4 that in the lab experiment, the errors of the photogrammetry system are bigger than that of the robot. This is because its typical value is bigger than the robot, that same as the simulation experiment shown in Section 3.3.1. The error of the photogrammetry system Δ*_CM_* in the lab experiment is smaller than the simulation test. This illustrates that the accuracy of the photogrammetry system in its effective FOV is improved, as we had tested in the repeatability experiment in Section 3.1.1. It is seen in Figure 8a,b and Table 4 that both of the fused methods can reduce the error after the data fusion. Through the data calculation in Table 4, the error is reduced by 67.3% with KF and by 38.2% with MOIFA. This is a little smaller than the results of the simulation, but both of them have common trends. However, the value of the fused error using KF is a little larger than the error of the simulation. The reason is that KF is a linear filter, which had been discussed previously in Section 3.3.1. Comparing to the other works, Yauheni and Jerzy of Warsaw University of Technology obtain the improved value of the positioning accuracy for the robot end-effector ΔL = 2.39 mm using the method of joint error mutual compensation. Li Junmin and Wang Jinge *et al.* improved the pose accuracy of the robot to 2.2 mm based on the unit quaternion and the prediction of the pose estimation accuracy. It is indicated that the multiple-sensor combination measuring system (MCMS) proposed in this paper has good performance for improving the pose accuracy of the robot.

Therefore, a conclusion can be made in comparison to the simulation that both of the data fusion methods can lead to the improvement of the results. MOIFA has more stable accuracy of the fusion, but it has no predicted function, which would cause hysteresis in the feedback control system of the robot. KF is widely applied in the areas of robotics and aviation. It possesses the predicted function of the next moment, which is suitable for real-time measurement and compensation. However, its predicted error would be enlarged with increasing measurement range, since it is a kind of linear filter. According to the features of the two data fusion methods, we can adopt KF to fuse data when dynamic and real-time measurement is needed, as well as when the measurement range is small. Otherwise, MOIFA can be adopted when the static and offline measurement is needed, as well as when the measurement range is large.

## 4. Conclusions

In this paper, we proposed a multi-sensor combination measuring system (MCMS) and two sensor data fusion methods to improve the pose accuracy of industrial robots. The advantage of this method is that it is automatic and does not involve environmental intervention. To ensure the accuracy of the measured sensor, this paper researched the repeatability precision of the photogrammetry system and the robot calibration by means of the photogrammetry system. The experimental results show that the best accuracy of the photogrammetry system is in the center of the FOV, which is the range of 1 × 0.8 × 1 ∼ 2 × 0.8 × 1 m. The position error of the robot manipulator is less than 1 mm after being calibrated by the photogrammetry system. In order to improve the accuracy of the robot pose, we propose two kinds of data fusion methods to fuse the redundant information gathered from the multiple sensors. Through comparing with the simulation and lab experimental results, KF possesses a predicted function of the next moment that is suitable for real-time measurement and compensation. However, its predicted error would be enlarged with an increasing measurement range, since it is a kind of linear filter. On the other hand, MOIFA possesses the stable accuracy of fusion, but it is not capable of the predicting function. This will cause hysteresis in the feedback control system of the robot. Therefore, both of the methods can reduce the pose error of the robot by 38%∼78%. The choice of method is dependent on the requirements of the measurement. The experimental and theoretical results provided the basis for an industrial application of the robot pose measurement and compensation. Future works will include the real-time transferring of data, online control and compensation for the pose of the robot manipulator.

## Figures and Tables

**Table 1:** The standard deviations of the repeated measurement of points (units: mm).

**Plane 1**	***δ****_x_*	***δ****_y_*	***δ*_z_**	***δ****_d_*
1	0.046	0.929	0.303	0.979
2	0.119	0.872	0.246	0.914
3	0.128	1.058	0.242	1.093
4	0.218	0.800	0.325	0.891
5	0.106	1.189	0.229	1.215
6	0.075	1.028	0.275	1.067
7	0.064	0.972	0.283	1.015
8	0.231	1.126	0.272	1.181

**Plane 5**	***δ****_x_*	***δ****_y_*	***δ****_z_*	***δ****_d_*

35	13.902	7.396	10.661	19.017
36	0.062	0.950	0.255	0.985
37	4.709	9.126	1.822	10.431
38	0.075	1.057	0.256	1.090
39	10.487	9.584	25.830	29.479
40	0.095	0.920	0.286	0.968
41	0.102	0.896	0.406	0.989
42	0.064	0.989	0.285	1.031
43	0.068	1.007	0.273	1.046

**Table 2:** The standard deviations of lines (units: mm).

**Plane 1**	***d****_l_*	***d****_c_*	**Δ*d***
1	400.202	400.249	−0.047
2	400.482	400.179	0.302
3	500.253	501.264	−1.010
4	500.140	499.492	0.647
5	400.794	400.350	0.443
6	400.509	400.491	0.018
7	500.325	500.498	−0.172
8	400.399	400.583	−0.184
9	500.112	500.306	−0.193

**Plane 5**	***d****_l_*	***d****_c_*	**Δ*d***

1	321.442	400.547	−79.104
2	398.357	401.150	−2.793
3	501.106	500.544	0.562
4	458.928	500.737	−41.809
5	409.822	401.110	8.711
6	401.108	400.693	0.415
7	499.526	500.038	−0.511
8	447.228	499.478	−52.249
9	499.533	500.300	−0.767
10	401.440	400.309	1.131
11	499.971	500.153	−0.181
12	401.508	402.316	−0.808

**Table 3:** Simulation results of the data fused by KF and MOIFA (units: mm).

**Δ*x*_1_**	**Δ*x*_2_**	**Δ*x****_f_*
−0.198	0.139	0.043
**Δ***_CM_*	**Δ***_RB_*	**Δ***_f_*
0.240	0.156	0.129

**Table 4:** Experimental results of the data fused by KF and MOIFA (units: mm).

**Δ*x*_1_**	**Δ*x*_2_**	**Δ*x****_f_*
−3.940	3.379	1.297
**Δ***_CM_*	**Δ***_RB_*	**Δ***_f_*
1.587	1.386	0.981
